# A New Scoring System Administered by Patients to Identify Moderate-to-Severe Chemotherapy-Induced Peripheral Neuropathy: Final Results of the NEURO-BREAC Trial

**DOI:** 10.3390/cancers18050835

**Published:** 2026-03-04

**Authors:** Dirk Rades, Maria Karolin Streubel, Christian Staackmann, Laura Doehring, Achim Rody, Maria Joy Normann Haverberg, Martin Ballegaard

**Affiliations:** 1 Department of Radiation Oncology, University of Lübeck, 23562 Lübeck, Germany; 2 Department of Radiation Oncology, Campus Lübeck, University Medical Center Schleswig-Holstein, 23538 Lübeck, Germany; mariakarolin.streubel@uksh.de (M.K.S.); christian.staackman@uksh.de (C.S.); laura.doehring@uksh.de (L.D.); 3 Department of Obstetrics and Gynecology, Campus Lübeck, University Medical Center Schleswig-Holstein, 23538 Lübeck, Germany; achim.rody@uksh.de; 4 Department of Neurology, Zealand University Hospital, 4000 Roskilde, Denmark; mjha@regionsjaelland.dk (M.J.N.H.); mbag@regionsjaelland.dk (M.B.); 5 Department of Clinical Medicine, Faculty of Health and Medical Sciences, University of Copenhagen, 2200 Copenhagen, Denmark

**Keywords:** breast cancer, chemotherapy, taxanes, peripheral neuropathy, scoring system

## Abstract

**Background/Objectives:** Many breast cancer patients treated with taxanes experience chemotherapy-induced peripheral neuropathy (CIPN). The early detection of CIPN may be facilitated by scoring systems. The existing Utah Early Neuropathy Scale (UENS) requires the presence of medical staff members. A self-assessment tool usable by patients is desired. Such an instrument was recently developed but had not yet been evaluated for the detection of CIPN. This prospective study aimed to identify the optimal cut-off score for the identification of moderate-to-severe CIPN in breast cancer survivors. **Methods:** Twenty-six breast cancer survivors (patients) who previously received taxane-based chemotherapy were included. Eighteen patients presenting with moderate-to-severe CIPN and eight patients without CIPN used the new scoring system (0–44 points). For each cut-off score, sensitivity, specificity, Youden index, and positive (PPV) and negative (NPV) predictive values were calculated. Patients rated their satisfaction with the tool. Dissatisfaction rates of >20% and >40%, respectively, would mean that it needed optimization or could not be used. Afterwards, the UENS (0–42 points) was applied by medical staff members. **Results:** For the new tool, a cut-off score of 9 points was found to be optimal for identifying moderate-to-severe CIPN. The sensitivity, specificity, Youden index, and PPV and NPV were 100% in each case. The dissatisfaction rate was 7.7%. When applying the UENS, the sensitivity, specificity, Youden index, and PPV and NPV were each 100% for a cut-off score of 6 points. **Conclusions:** The new self-assessment scoring system was highly accurate regarding the identification of moderate-to-severe CIPN. Patient satisfaction was high. When considering the limitations of this trial, the new instrument may be used in future studies.

## Simple Summary

Breast cancer patients receiving taxane-based treatment often develop chemotherapy-induced peripheral neuropathy (CIPN). Since treatment options for CIPN are very limited, its early diagnosis appears important. This may be facilitated by scoring systems. The existing objective systems need to be applied by staff members. A tool that can be used by the patients themselves is desirable. Such an instrument was recently developed but not tested for the detection of CIPN. The definition of the most appropriate cut-off score to identify moderate-to-severe CIPN is an important step for the evaluation of the usability of the scoring system in patients with CIPN. In this prospective trial performed on breast cancer survivors, the optimal cut-off score was identified. It provided extremely high accuracy, achieving the maximum possible Youden index of 1.00. Moreover, patient satisfaction with the new scoring system was very high. Given the limitations of this study, the new instrument may be used in future studies.

## 1. Introduction

Neoadjuvant or adjuvant chemotherapy is indicated in a considerable number of patients with non-metastatic breast cancer [1]. Very often, the chemotherapy regimen includes a taxane, namely, paclitaxel or docetaxel. These agents are known to be associated with a considerable risk of “chemotherapy-induced peripheral neuropathy” (CIPN) [2]. This adverse event leads to relevant symptoms including sensory disturbances (numbness), paresthesia (tingling), pain, and motor symptoms (impairment of fine motor skills and weakness), predominantly in the lower and upper extremities. CIPN can last comparably long and have a significantly negative effect on a patient’s quality of life and capacity to cope with everyday life, particularly if the symptoms are severe (grade 3) or at least moderate (grade 2) [3,4,5,6,7,8,9]. Unfortunately, the options for both prophylactic treatment and treatment of existing CIPN are limited. In a review article that covered different aspects of CIPN including its treatment and prevention, it was stated that there are no preventative therapies for this complication [2]. A similar statement can be found in another review article from 2019 [10]. In addition, an updated practice guideline of the American Society of Clinical Oncology does not recommend any agent for the prophylactic treatment of CIPN [11,12]. Moreover, according to this updated guideline, duloxetine was the only drug that showed some evidence that it may be used for the treatment of existing CIPN [12]. In a randomized trial, duloxetine was shown to be effective at improving pain as a symptom of CIPN and improving numbness and tingling of the feet (but not of the hands) [13]. The early detection of CIPN and its potential modification appear important in order to decrease the risk of progression and the development of long-term CIPN [6,7,8,14,15,16,17]. The identification of CIPN would likely be facilitated by the use of scoring systems. The available scoring systems include the Utah Early Neuropathy Scale (UENS) and the Total Neuropathy Score (TNS) [18,19,20,21,22,23,24,25,26]. However, these tools need to be applied by trained medical staff members and require specific equipment including a 1¾ inch safety pin, a 128 Hz tuning fork, and a reflex hammer. Thus, a scoring system that can be applied by the patients themselves without the presence of medical staff members would be welcomed. Such a tool for self-assessment by a patient has been recently developed but had not yet been evaluated for the detection of CIPN [27,28]. One of the initial steps to determine its value for cancer patients is the identification of the optimal cut-off score for differentiation between no CIPN and moderate-to-severe CIPN. This prospective trial primarily aimed to identify the optimal cut-off score in a cohort of breast cancer survivors who were previously treated with paclitaxel- or docetaxel-based chemotherapy for non-metastatic disease [29].

## 2. Materials and Methods

**Figure 1:**
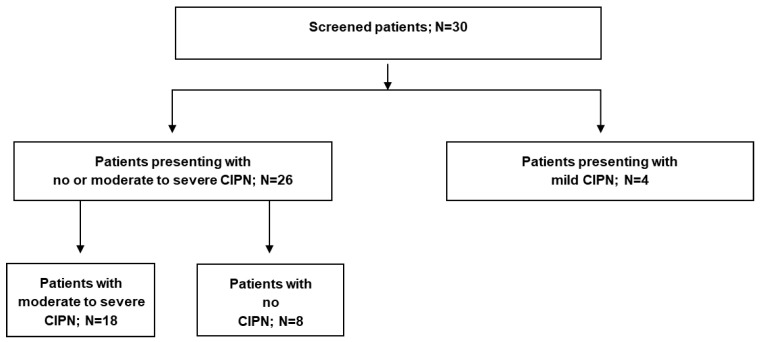
Patient enrolment and eligibility for the primary objective.

A total of 26 female breast cancer survivors (patients) were included in the prospective interventional NEURO-BREAC trial (Figure 1), which received approval from ethics committees (EC) in Lübeck (leading EC, code 2025-339_2) and Hannover and is registered at clinicaltrials.gov (NCT07148336).

### 2.1. Study Participants

In accordance with the study protocol and the ethics approval, the participants of this trial were mainly recruited from a cohort of breast cancer patients evaluated for CIPN in a previous retrospective study [30]. They had received chemotherapy including paclitaxel or docetaxel and adjuvant irradiation and had no or moderate-to-severe CIPN. The median interval between the last day of chemotherapy and the day of the investigations for the NEURO-BREAC trial was 36 months (first quartile = 28 months and third quartile = 40 months). The chemotherapy regimens included epirubicin/cyclophosphamide plus paclitaxel (EC + PAC), epirubicin/cyclophosphamide plus paclitaxel/carboplatin (EC + PAC/Carbo), epirubicin/cyclophosphamide plus paclitaxel/carboplatin plus pembrolizumab (EC + PAC/Carbo + Pembro), epirubicin plus paclitaxel plus cyclophosphamide (ETC), and docetaxel plus carboplatin plus trastuzumab plus pertuzumab (TCbHP) [30,31]. Additional patient characteristics are shown in Table 1.

### 2.2. Study Procedures

The trial was conducted to contribute to the evaluation of the value of a new scoring system for the detection of CIPN. The main objective was the identification of the optimal cut-off score to differentiate between no CIPN and moderate-to-severe CIPN. The grade of CIPN was determined according to the clinical section of the TNS [23,24,25,26]. Based on the CIPN-related symptoms and signs (sensory, motor, and autonomic deficits and reduced pin sensitivity, vibration sensibility, and reflexes) and their grade (ranging between normal = 0 points and very pronounced = 4 points), the possible patient scores were between 0 and 28 points. In the NEURO-BREAC trial, no CIPN was defined as 0–1 point and moderate-to-severe CIPN as ≥8 points [26]. According to this definition, 18 patients had moderate-to-severe CIPN and 8 patients had no CIPN, respectively.

The 26 patients used the new self-administered scoring system, which was supported by a neuropathy tracker [27,28]. The neuropathy tracker is a mobile health tool that can be self-administered by patients. The patients were asked to indicate and rate their symptoms related to CIPN and were guided through an evaluation of a pinprick sensation (using a safety pin) and vibration (using the patient’s mobile phone) at several regions of both feet and their lower legs. In addition, motor strength (dorsal extension of the big toes) was evaluated by the patients.

The scoring system included general symptoms (0–4 points), pin-prick sensation in six pre-defined regions of each leg (0–24 points), allodynia/hyperalgesia (0–4 points), two aspects of large fiber sensation (0–6 points and 0–2 points, respectively), and motor function (0–4 points). Thus, the possible patient scores ranged between 0 and 44 points.

### 2.3. Statistical Considerations

The trial aimed to determine the cut-off score associated with the highest accuracy for predicting moderate-to-severe CIPN. Sensitivity and specificity were calculated for every patient score. The relation between sensitivity and specificity was illustrated using a receiver operating characteristic (ROC) curve and the area under the curve (AUC). The ROC was defined as the plot of sensitivity versus 1-specificity (false-positive rate) across varying cut-offs. An ROC curve located closer to the upper-left-hand corner would correspond to a greater discriminant capacity of the symptom-based scoring system. The AUC summarized the entire location of the ROC curve. In the case of an AUC of 1.0, the cut-off score was considered perfect. In the case of a value of ≤0.7, the score was considered not useful [32]. Moreover, a cut-off score with a sensitivity of at least 90% and a specificity of at least 80% was considered optimal. In addition, the Youden index (sensitivity + specificity − 1), the positive predictive value (PPV), and the negative predictive value (NPV) were calculated to suggest an optimal cut-off score.

An additional sensitivity analysis was performed considering the relationship between the tertiles of the scores and the occurrence of moderate-to-severe CIPN. For this analysis, the Jonckheere–Terpstra test was used, which tested the global null hypothesis that the distribution of the response variable did not differ between the tertiles. This test should have identified whether the incidence of moderate-to-severe CIPN increased with the tertiles of the scores. The test specifically evaluated whether there was a “monotonic trend” in the frequency of moderate-to-severe CIPN across increasing scores, reflecting the hypothesis that higher scores were associated with higher rates of CIPN. If no monotonic trend was identified, the clinical benefit of the scoring system would be questioned.

To include an instrument often used in studies evaluating peripheral neuropathy and to obtain “objective” results from an investigation performed by a medical staff member, the UENS was applied. For the UENS, the same parameters of accuracy were calculated as for the new scoring system.

### 2.4. Additional Analyses

In addition, the potential impacts of patient-, tumor-, and treatment-related characteristics on the rating of moderate-to-severe CIPN were investigated. These characteristics are shown in Table 1. For the corresponding analyses, Fisher’s exact tests were applied. A *p*-value < 0.05 was considered significant. Statistical analyses for the NEURO-BREAC trial were performed with version 9.4. of the SAS software (SAS Institute Inc., Cary, NC, USA).

Moreover, patient satisfaction with the new scoring system was evaluated. The patients were asked to complete a questionnaire [33,34]. They rated separate scales ranging from 1 to 7 points whether they considered the new tool comprehensible, easy to handle, helpful, and stabilizing (four categories). Higher scores represented a higher degree of satisfaction. The mean scores plus standard deviations were calculated for each category. In addition, the mean score was calculated for each patient. Patients were rated as not satisfied if the mean score was <4.0 [35]. The percentage of patients who were not satisfied would have a consequence for the potential further use of the new scoring system. If the satisfaction rate was <80%, the scoring system would need improvement, and if the rate was <60%, it would not be suitable for future clinical trials.

## 3. Results

The scores obtained with the new scoring system ranged between 0 and 30 points. In the entire cohort, the mean score (plus standard deviation) was 13.57 (±8.76) points, and the median score (plus range) was 14.5 (0.0–30.0) points.

In the 18 patients with moderate-to-severe CIPN, the mean and median scores were 18.39 (±5.53) points and 16.5 (9.0–30.0) points, respectively. Two of these patients received symptomatic treatment with pregabalin and one patient with pregabalin and duloxetine. The scores for these three patients were 16 points, 20 points, and 26 points, respectively. Other diseases that may have caused peripheral neuropathy, e.g., diabetes or alcoholic neuropathy, were not reported by any patient with moderate-to-severe CIPN. In patients with no CIPN, the mean and median scores were 2.75 (±2.55) points and 3.5 (0.0–7.0) points, respectively. No patient in this group received analgesics or had a disease that may have led to peripheral neuropathy.

**Figure 2:**
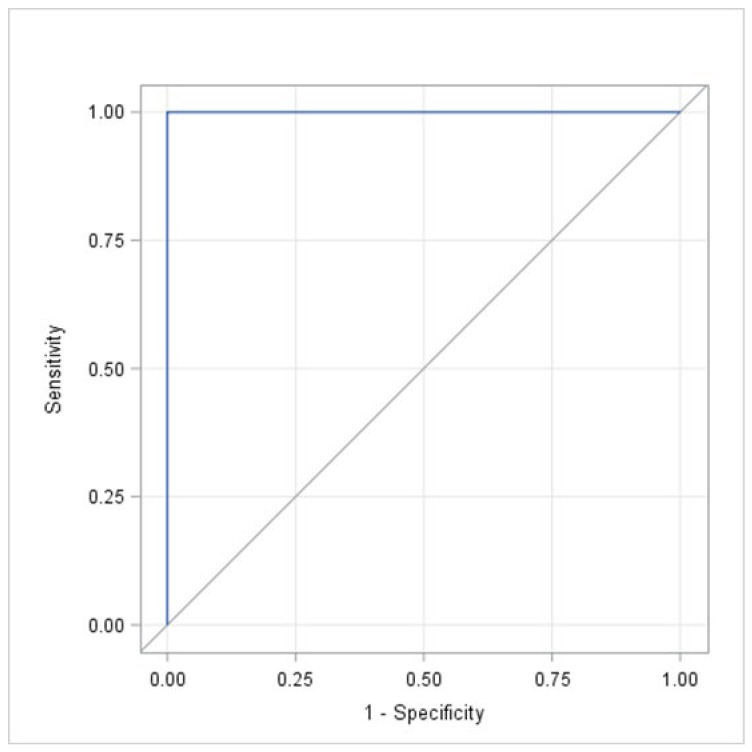
The receiver operating characteristic (ROC) curve used to illustrate the relationship between sensitivity and specificity, defined as the plot of sensitivity versus 1-specificity (false-positive rate) across the different cut-off values when using the new scoring system.

The sensitivity, specificity, Youden index, and PPV and NPV for each obtained score are summarized in Table 2. According to the results presented in Table 2, a cut-off score of 9 points provided the highest accuracy, with all five parameters being 100% (Youden index = 1.00). This result was supported by the ROC curve shown in Figure 2. The observed AUC was 1.00 with a 95% confidence interval of [1.00; 1.00].

Initially, we aimed to estimate the area under the ROC curve (AUC) and its 95% confidence interval for a diagnostic test distinguishing patients with no CIPN and patients with moderate-to-severe CIPN. Initially, we planned to apply the nonparametric variance estimation used by DeLong et al. [36], which is widely recommended for AUC inference. However, the scores of the new scoring system showed complete separation between the groups, resulting in an AUC of 1.0 and zero variance under DeLong’s method, producing an uninformative confidence interval of [1.00, 1.00], as shown above. To address this, we used an exact approach based on the binomial interpretation of AUC as the probability that randomly selected patients with moderate-to-severe CIPN had scores that were higher than a patient with no CIPN. Thus, the 95% confidence interval was computed using the Clopper–Pearson exact interval for a binomial proportion, with the lower bound derived from the beta distribution quantile and the upper bound fixed at 1.0. This method provides a conservative yet informative interval in cases of complete separation. Consequently, the resulting 95% confidence interval for the AUC was [0.975; 1]. The test, whether the AUC was greater than 0.7, yielded statistical significance, with *p* < 0.0001.

Moreover, in the additional sensitivity analysis (scores stratified by tertiles) performed with the Jonckheere–Terpstra test, the rates of moderate-to-severe CIPN were 11% (one of in nine patients), 100% (eight of eight patients), and 100% (nine of 9 patients) in patients with ≤9 points, 10–16 points, and ≥17 points, respectively (*p* < 0.0001).

**Figure 3:**
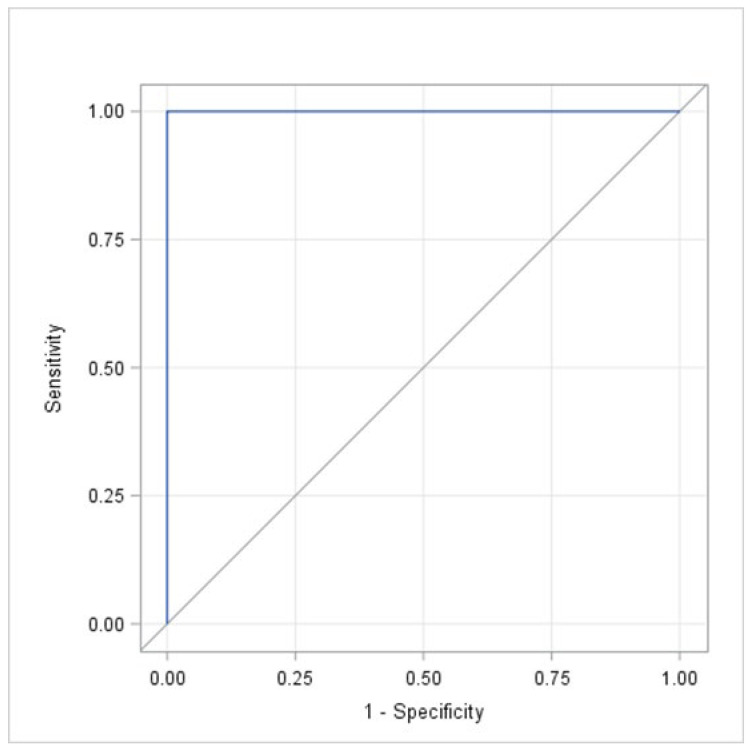
The receiver operating characteristic (ROC) curve used to illustrate the relationship between sensitivity and specificity, defined as the plot of sensitivity versus 1-specificity (false-positive rate) across the different cut-off values when using the Utah Early Neuropathy Scale (UENS).

**Figure 4:**
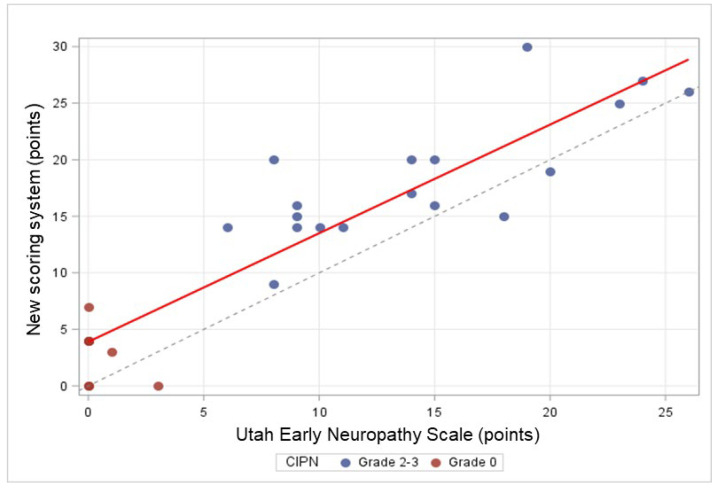
Comparison of the new scoring system and the Utah Early Neuropathy Scale (UENS) with respect to the distribution of patients with no CIPN and those with moderate-to-severe CIPN.

When applying the UENS, the scores ranged between 0 and 26 points. In the entire cohort, the mean score was 10.08 (±8.26) points, and the median score was 9.0 (0.0–26.0) points. The mean scores were 14.33 (±6.13) points in the patients with moderate-to-severe CIPN and 0.50 (±1.07) points in patients with no CIPN, respectively. The median scores were 14.0 (6.0–26.0) points and 0.0 (0.0–3.0) points, respectively. The sensitivity, specificity, Youden index, PPV, and NPV for each score obtained with the UENS are summarized in Table 3. According to these results, a cut-off score of 6 points provided the highest accuracy when applying the UENS, with all five parameters being 100% (Youden index = 1.00). The observed AUC of the ROC curve was 1.00 with a 95% confidence interval of [1.00; 1.00] (Figure 3). The more relevant exact 95% confidence interval for the AUC, obtained by using the Clopper–Pearson exact interval for a binomial proportion, was [0.975; 1]. As with the new scoring system, the test, whether the AUC was greater than 0.7, demonstrated statistical significance (*p* < 0.0001). The accuracy of the new scoring system and the UENS appeared comparable, which is shown in Figure 4. The linear and monotonic association between the two scores was assessed through Pearson’s and Spearman rank-order correlation measures, leading to 0.90 and 0.88, respectively.

In the analysis regarding the potential impacts of patient-, tumor-, and treatment-related characteristics, a lower Karnofsky performance score of 70–80 (*p* = 0.0001), a history of hypertension (*p* = 0.0095), and beta blocker treatment (*p* = 0.023) were significantly associated with the occurrence of moderate-to-severe CIPN. In addition, a trend was found for a BMI of ≥30 kg/m^2^ (*p* = 0.062). The results of all characteristics are shown in Table 1.

In addition, patients rated their satisfaction with the new scoring system. The mean scores of individual patients were <4.00 for two patients (7.7%), 4.00–4.90 for no patients (0.0%), 5.00–5.90 for five patients (19.2%), 6.00–6.90 for 11 patients (42.3%), and 7.00 for eight patients (30.8%), respectively. As 24 patients had a mean score of ≥4.0 points, the rate of satisfaction was 92.3%, i.e., it was well above the required 80%. The median patient score was 6.375 (5.75–7.00) points. The mean scores (plus standard deviations) of the four categories (comprehensible, easy to handle, helpful, and stabilizing) are given in Table 4.

## 4. Discussion

Patients with breast cancer receiving paclitaxel- or docetaxel-based chemotherapy may develop moderate-to-severe CIPN, which, due to its symptoms including neurologic deficits and pain, can have a demonstrable negative effect on a patient’s everyday life [2]. The risk of CIPN is likely further increased if the chemotherapy regimen also includes platin derivates [37]. Since treatment options for symptoms of CIPN other than pain are extremely limited and not proven in prospective trials, it appears important to identify this adverse event very soon and adapt the treatment protocol [6,7,8,11,12,13,14,15,16,17]. Moreover, close monitoring of patients treated with taxane-based or platin-based chemotherapy is recommended during the time of chemotherapy and the follow-up period. Both the early identification of CIPN and the monitoring of patients at risk can be improved by the application of scoring instruments. Existing objective instruments such as the UENS and TNS need to be performed by medical staff members and require special neurologic equipment [18,19,20,21,22,23,24,25,26]. To further facilitate both diagnosis and monitoring, an instrument eligible for self-assessment by patients at home would be ideal. A potentially suitable scoring system supported by a neuropathy tracker was recently developed in Denmark for the detection of peripheral neuropathy in general [27,28]. Depending on the presence and severity of neuropathy-related symptoms, patients are assigned 0 to 44 points (higher score = higher degree of neuropathy). However, this tool had not yet been evaluated in breast cancer patients with CIPN. To be properly used in this patient group, the optimal cut-off score for discrimination between the absence and presence of CIPN needed to be defined. The first step would be to define the optimal cut-off score for discrimination between no and moderate-to-severe CIPN. If this was not possible, further studies on breast cancer patients with mild CIPN would not appear reasonable. Therefore, the present prospective NEUR-BREAC trial was performed on breast cancer patients with either no CIPN or moderate-to-severe CIPN.

According to its results, a cut-off score of 9 points provided the highest accuracy, with a sensitivity of 100% and a specificity of 100%, resulting in the maximum possible Youden index of 1.00. Thus, two requirements that were pre-specified in the study protocol of the NEURO-BREAC trial [29] were met, namely, a sensitivity of at least 90% and a specificity of at least 80%. The requirements were also met when using the UENS. The accuracy of both tools appeared comparable. Moreover, patient satisfaction with the new scoring system was assessed. The satisfaction rate was very high (92.3%). Therefore, the new scoring system may be used in future clinical trials.

### Limitations

When aiming to use the new scoring system, the limitations of the NEURO-BREAC trial should be considered. One must be aware that this trial was designed to provide initial evidence on the performance of the new scoring system. Moreover, its results apply only for the prevalence of moderate-to-severe CIPN in the specific dataset of this trial. In a small, mono-centric study like the NEURO-BREAC trial, this prevalence cannot be estimated without a selection bias. Another possible limitation caused by the limited sample size is the fact that no patient had a score of 8 points or between 10 and 13 points. Thus, one or more of these scores might also be associated with a Youden index of 1.00. Moreover, the results found in a cohort of breast cancer patients treated with paclitaxel- or docetaxel-based chemotherapy and adjuvant irradiation may apply to patients receiving a different treatment regimen or patients with metastatic disease. Since the gap between the maximum score for patients with no CIPN (7 points) and the minimum score for patients with moderate-to-severe CIPN (9 points) was very small, it appears questionable whether the score may be useful for the identification of mild CIPN. The same limitations apply to the UENS. Therefore, the new scoring tool likely needs to be refined. An updated version of the tool may include more sections investigated with respect to pinprick sensation, more than three points per leg where the vibration is tested, a higher score for painful sensation to prick or touch, the assessment of additional neurologic symptoms, and a higher score for the evaluation of motor strength. These modifications will result in a higher total score, which may allow for a better differentiation between no CIPN and mild CIPN and between mild CIPN and moderate-to-severe CIPN. Additional studies are required to obtain an appropriate scoring tool for the early detection of CIPN, when it is still mild.

According to the results of the NEURO-BREAC trial, a lower Karnofsky performance score of 70–80, a history of hypertension, and beta blocker treatment were significantly associated with moderate-to-severe CIPN, and a BMI of ≥30.0 kg/m^2^ showed a trend. The prognostic value of these characteristics was also observed in previous studies. In the studies by Lixian et al. and Hiramoto et al., a history of hypertension was an independent risk factor of CIPN in breast cancer patients treated with taxane-based chemotherapy [38,39]. In two other studies that evaluated the potential risk factors of CIPN in breast cancer patients receiving taxane-based chemotherapy, significant associations between CIPN and beta blocker treatment were observed [40,41]. Moreover, in a retrospective study of 503 patients, for the 413 patients with breast cancer and 90 patients with ovarian cancer, treatment with beta blockers was identified as a risk factor of CIPN [42]. Data supporting the prognostic role of the Karnofsky performance score are scarce. Such an association was shown only in our previous retrospective study [30]. However, in a review article, concomitant diseases were suggested to increase the risk of CIPN [42]. One may speculate that the overall burden of concomitant diseases had a negative impact on a patient’s performance score. Although the impact of a higher BMI on the occurrence of CIPN showed only a trend in the present trial, its prognostic role should not be underestimated. A considerable number of studies was identified in the literature to have found a significant association between a higher BMI and CIPN [6,38,39,40,43,44,45,46,47,48,49,50,51]. Despite the fact that the data of the NEURO-BREAC trial regarding the risk factors of CIPN mainly agree with the results of previous studies, which suggests consistency in our findings, the above-mentioned limitations also need to be considered during the interpretation in this context.

## 5. Conclusions

For both the new self-assessment scoring system and the objective UENS, the optimal cut-off score for discriminating between no CIPN and moderate-to-severe CIPN was identified. Both tools were highly accurate regarding the identification of moderate-to-severe CIPN, achieving the maximum possible Youden index of 1.00. Moreover, patient satisfaction with the new scoring system was very high. Thus, when considering the limitations of the present trial, the new scoring system may be used in future clinical trials.

## Figures and Tables

**Table 1:** Patient-, tumor-, and treatment-related characteristics of the 26 patients eligible for the primary endpoint. The *p*-values were obtained from Fisher’s exact tests.

	Moderate or Severe CIPN, n (%)	*p*-Value
**Age** 65 years (*n* = 21) ≥65 years (*n* = 5)	13 (62) 5 (100)	0.28
**Karnofsky performance score** 90–100 (*n* = 11) 70–80 (*n* = 15)	3 (27) 15 (100)	0.0001
**Body mass index** <30 kg/m^2^ (*n* = 19) ≥30 kg/m^2^ (*n* = 7)	11 (58) 7 (100)	0.062
**Autoimmune disease** No (*n* = 19) Yes (*n* = 7)	14 (74) 4 (57)	0.64
**Cardiovascular disease** No (*n* = 23) Yes (*n* = 3)	15 (65) 3 (100)	0.53
**Hypertension** No (*n* = 16) Yes (*n* = 10)	8 (50) 10 (100)	0.0095
**Smoking history** <10 pack years (*n* = 18) ≥10 pack years (*n* = 8)	11 (61) 7 (88)	0.36
**Current Smoker** No (*n* = 21) Yes (*n* = 5)	13 (62) 5 (100)	0.28
**Beta blocker treatment** No (*n* = 17) Yes (*n* = 9)	9 (53) 9 (100)	0.023
**Tumor histology** No special type alone (*n* = 20) Others (*n* = 5)	13 (65) 5 (83)	0.63
**Primary tumor stage** T1 (*n* = 7) T2 or T3 (*n* = 19)	4 (57) 14 (74)	0.64
**Nodal stage** N0 (*n* = 10) N+ (*n* = 16)	9 (90) 9 (56)	0.10
**Type of surgery** Breast conserving surgery (*n* = 19) Mastectomy (*n* = 7)	15 (79) 3 (43)	0.15
**Axillary lymph node dissection** No (*n* = 10) Yes (*n* = 16)	9 (90) 9 (56)	0.10
**Type of chemotherapy** EC + PAC (*n* = 13 EC + PAC/Carbo (*n* = 4) EC + PAC/Carbo + Pembro (*n* = 5) ETC (*n* = 1) TCbHP (*n* = 3)	10 (77) 2 (50) 3 (60) 1 (100) 2 (67)	0.85
**Carboplatin** No (*n* = 14) Yes (*n* = 12)	11 (79) 7 (58)	0.40
**Timing of chemotherapy** Neoadjuvant (*n* = 17) Adjuvant (*n* = 9)	10 (58) 8 (89)	0.19
**Current hormonal therapy** No (*n* = 8) Yes (*n* = 18)	5 (63) 13 (72)	0.67

**Table 2:** Estimation of the sensitivities, specificities, Youden indices, positive predictive values (PPV), and negative predictive values (NPV) stratified by the specific cut-offs for the new scoring system.

Cut-Off Score	Sensitivity (%)	Specificity (%)	Youden Index (%)	PPV (%)	NPV (%)
**30 points**	5.56	100.0	5.56	100.0	32.00
**27 points**	11.11	100.0	11.11	100.0	33.33
**26 points**	16.67	100.0	16.67	100.0	34.78
**25 points**	22.22	100.0	22.22	100.0	36.36
**20 points**	38.89	100.0	38.89	100.0	42.11
**19 points**	44.44	100.0	44.44	100.0	44.44
**17 points**	50.00	100.0	50.00	100.0	47.06
**16 points**	61.11	100.0	61.11	100.0	53.33
**15 points**	72.22	100.0	72.22	100.0	61.54
**14 points**	94.44	100.0	94.44	100.0	88.89
**9 points**	100.0	100.0	100.0	100.0	100.0
**7 points**	100.0	87.50	87.50	94.74	100.0
**4 points**	100.0	50.00	50.00	81.82	100.0
**3 points**	100.0	37.50	37.50	78.26	100.0
**0 points**	100.0	0.00	0.00	69.23	

**Table 3:** Estimation of sensitivities, specificities, Youden-indices, positive predictive values (PPV), and negative predictive values (NPV) stratified by the specific cut-offs for the Utah Early Neuropathy Scale (UENS).

Cut-Off Score	Sensitivity (%)	Specificity (%)	Youden Index (%)	PPV (%)	NPV (%)
**26 points**	5.56	100.0	5.56	100.0	32.00
**24 points**	11.11	100.0	11.11	100.0	33.33
**23 points**	16.67	100.0	16.67	100.0	34.78
**20 points**	22.22	100.0	22.22	100.0	36.36
**19 points**	27.78	100.0	27.78	100.0	38.10
**18 points**	33.33	100.0	33.33	100.0	40.00
**15 points**	44.44	100.0	44.44	100.0	44.44
**14 points**	55.56	100.0	55.56	100.0	50.00
**11 points**	61.11	100.0	61.11	100.0	53.33
**10 points**	66.67	100.0	66.67	100.0	57.14
**9 points**	83.33	100.0	83.33	100.0	72.73
**8 points**	94.44	100.0	94.44	100.0	88.89
**6 points**	100.0	100.0	100.0	100.0	100.0
**3 points**	100.0	87.50	87.50	94.74	100.0
**1 point**	100.0	75.00	75.00	90.00	100.0
**0 points**	100.0	0.00	0.00	69.23	

**Table 4:** Patient satisfaction with the new scoring system. For each category, 1 to 7 points could be assigned, and higher scores represented higher degrees of satisfaction.

Symptom-Based Score Was Considered	Mean Score	Standard Variation
Comprehensible	6.27	1.51
Easy to handle	6.00	1.44
Helpful	6.50	0.97
Stabilizing	5.88	1.50

## Data Availability

Further information regarding this trial is available at clinicaltrials.gov (identifier: NCT07148336, registered on 28 August 2025).

## Abbreviations

The following abbreviations are used in this manuscript:

AUC	Area under the curve
BMI	Body-mass index
CIPN	Chemotherapy-induced peripheral neuropathy
EC	Epirubicin/cyclophosphamide
ETC	Epirubicin plus paclitaxel plus cyclophosphamide
NPV	Negative predictive value
PAC	Paclitaxel
Pembro	Pembrolizumab
PPV	Positive predictive value
ROC	Receiver operating characteristic
TCbHP	Docetaxel plus carboplatin plus trastuzumab plus pertuzumab
TNS	Total Neuropathy Score
UENS	Utah Early Neuropathy Scale
